# Enzyme-independent catabolism of cysteine with pyridoxal-5′-phosphate

**DOI:** 10.1038/s41598-022-26966-6

**Published:** 2023-01-06

**Authors:** Prajakatta Mulay, Cindy Chen, Vijay Krishna

**Affiliations:** 1 Department of Biomedical Engineering, Lerner Research Institute, Cleveland Clinic, Cleveland, OH 44195 USA; 2 Department of Biomedical Engineering, Cleveland Clinic Lerner College of Medicine, Case Western Reserve University, Cleveland, OH 44106 USA

**Keywords:** Biochemistry, Biochemistry, Catalysis, Chemical biology, Origin of life

## Abstract

Pyridoxal-5′-phosphate (PLP) is a versatile cofactor that assists in different types of enzymatic reactions. PLP has also been reported to react with substrates and catalyze some of these reactions independent of enzymes. One such catalytic reaction is the breakdown of cysteine to produce hydrogen sulfide (H_2_S) in the presence of multivalent metal ions. However, the enzyme-independent catalytic activity of PLP in catabolizing cysteine in the absence of multivalent ions is unknown. In this study, we show that PLP reacts with cysteine to form a thiazolidine product, which is supported by quantum chemical calculations of the absorption spectrum. The reaction of PLP with cysteine is dependent on ionic strength and pH. The thiazolidine product slowly decomposes to produce H_2_S and the PLP regenerates to its active form with longer reaction times (> 24 h), suggesting that PLP can act as a catalyst. We propose an enzyme-independent plausible reaction mechanism for PLP catalyzed cysteine breakdown to produce H_2_S, which proceeds through the formation of thiazolidine ring intermediates that later hydrolyzes slowly to regenerate the PLP. This work demonstrates that PLP catalyzes cysteine breakdown in the absence of enzymes, base, and multivalent metal ions to produce H_2_S.

## Introduction

Discovered in 1942 by Snell et al. pyridoxal-5′-phosphate (PLP) is the metabolically active form of Vitamin B_6_. It is one of the most versatile cofactor for enzymes that catalyze a multitude of reactions with amino acids^1^. PLP as a cofactor is used in nearly 4% of all enzyme activities^2^. PLP-dependent enzymes catalyze transaminations, racemizations, decarboxylations, α, β, and γ-substitutions and eliminations, transaldolations, Claisen condensations, and more recently, oxidative deaminations in the presence of amino acids^3^. Interestingly, PLP is also able to catalyze some of these reactions in an enzyme-independent manner. Transaminations, α, β—substitutions, and decarboxylations with amino acids are catalyzed by PLP and multivalent metal ions non-enzymatically in aqueous solutions^4–11^, although they have also been reported to proceed slowly without metal ions^12^. For example, PLP in the absence of enzymes undergoes slow transamination when heated with amino acids to produce pyridoxamine^4^. These studies concluded that the aldehyde group of PLP acts as the active site and reacts readily and reversibly with amino acids to form Schiff bases, which depending on other reaction conditions, react further to form products. Since PLP generates these products independent of the enzyme, it is important to understand the direct interaction of PLP with essential amino acids.

**Figure 1:**
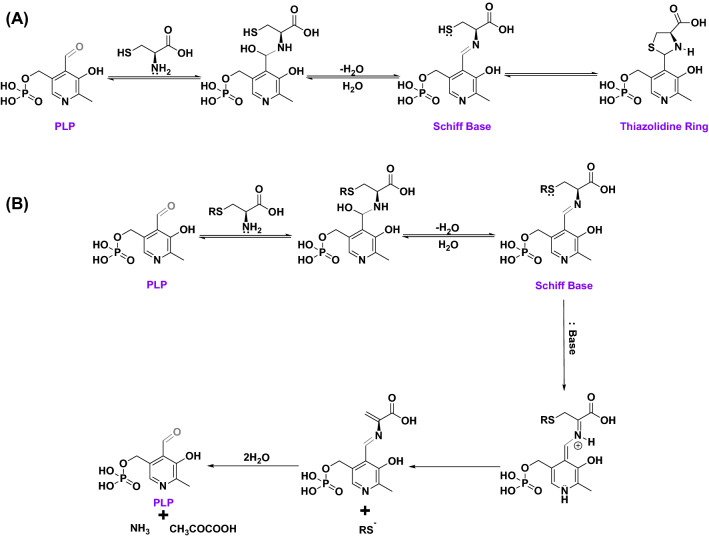
Proposed reaction mechanism of enzyme-independent chemical interaction of PLP with cysteine. **(A)** Mechanism of the formation of thiazolidine ring with PLP and cysteine in the absence of a base and metal ions. Formation of Schiff base is an intermediate step. **(B)** Mechanism for the catalytic behavior of PLP on reaction with *S*-substituted cysteines in slightly alkaline conditions. PLP is regenerated producing *S*-substituted analogs, ammonia, and pyruvate.

The enzyme-independent catalytic role of PLP on reaction with cysteine in the absence of multivalent metal ions has not been explored yet. This is because the reaction of PLP with cysteine has been widely reported to form a stable product, a thiazolidine ring, via condensation^13–17^. Early studies concluded that the thiazolidine ring formation proceeds in three steps: first the addition of amine group from cysteine on the aldehyde group of the PLP, second the removal of water to form an aldiminic Schiff base, and third ring closure (Fig. 1A). The thiazolidine ring was reported to be stable for more than 24 h, however, longer studies were not reported^15^. Alternatively, the thiol group from cysteine can also react with the aldehyde group of the PLP to form a hemimercaptal or mercaptal intermediate^17^. Interestingly, when cysteine derivatives such as *S*-(*p*-substituted phenyl) cysteines were reacted with PLP in slightly alkaline conditions, they undergo α, β—eliminations to produce ammonia, pyruvate, and *S*-(*p*-substituted phenyl) analogs and the PLP is regenerated (Fig. 1B). This confirmed the catalytic role of PLP in *S*-substituted cysteine breakdown in slightly alkaline conditions^18,19^.

Recently, Hine et al. investigated the non-enzymatic role of PLP in cysteine break down to produce hydrogen sulfide (H_2_S) in the presence of trivalent metal ions^9^. Endogenous H_2_S is predominantly produced from cysteine and homocysteine by enzymes such as cystathionine-β-synthase (CBS), cystathionine-γ-lyase (CGL), and 3-mercaptopyruvate sulfur-transferase (3-MST) with PLP as the cofactor^20^. H_2_S is an essential nutrient for the body and deficiencies in endogenous enzymatic H_2_S production or activity of CBS, CGL, and 3-MST are associated with several detrimental health effects including neurodegenerative disorders^21,22^. Thus, investigating the enzyme-independent catalytic role of PLP in cysteine breakdown becomes imminent to assess its capability in rescuing potential enzyme deficiencies responsible for the progression of neurodegenerative disorders.

Current literature on the enzyme-independent catalytic role of PLP in metabolizing cysteine lacks investigation of this interaction at physiological conditions. The catalytic role of PLP in the presence of either a base or multivalent metal ions is well established, however, the time-dependent catalytic behavior of PLP in the absence of both needs to be determined for a holistic understanding of the PLP interactions with cysteine. Therefore, in this study, we investigated the interactions of PLP with cysteine at physiological conditions. ^1^H-NMR and UV–Vis spectroscopy were used to monitor the progress of the reaction. Paper chromatography was performed to monitor the evolution of the product H_2_S gas. Quantum chemical calculations were performed on the probable intermediates to predict their UV–Vis absorption and compared with experimental data.

## Results and discussion

**Figure 2:**
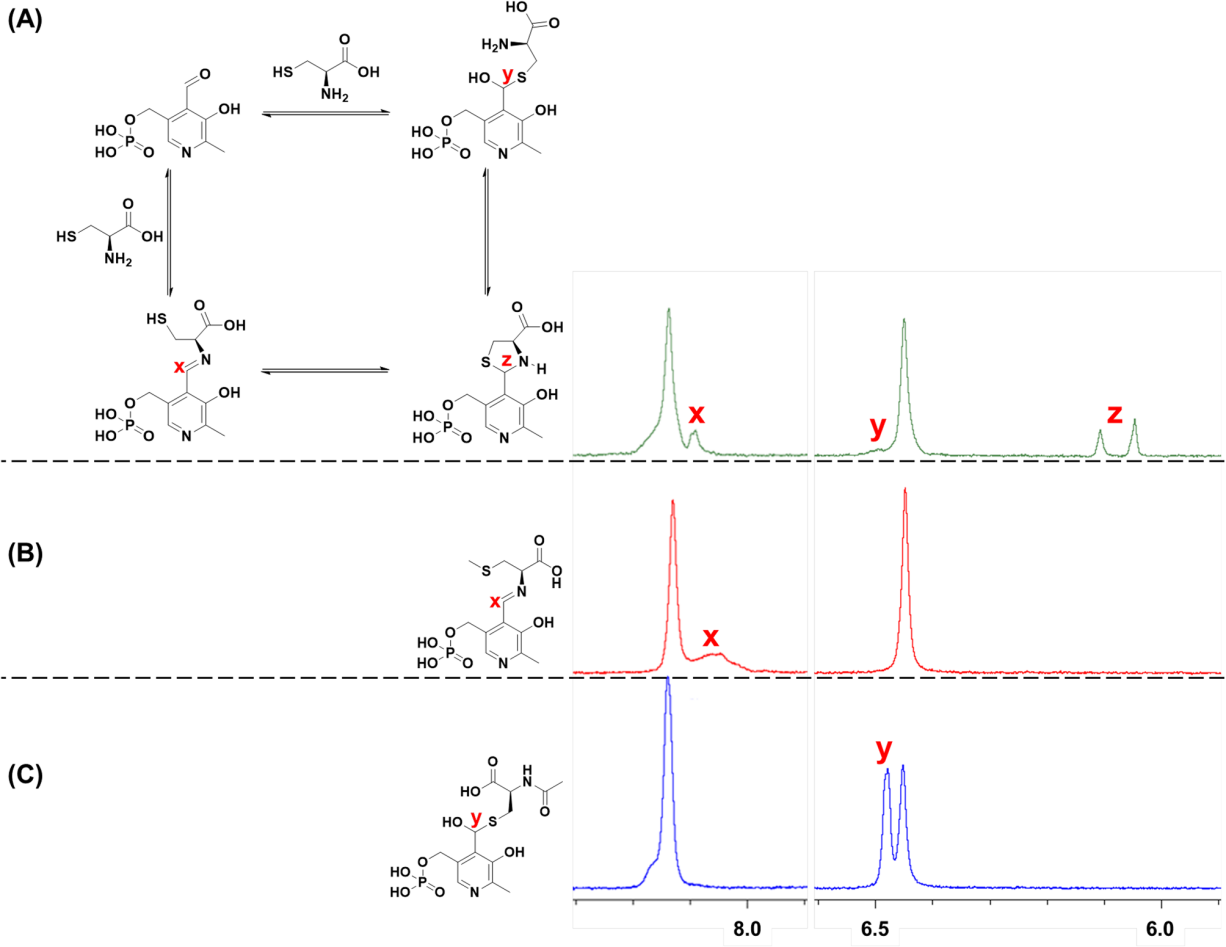
^1^H-NMR zoomed spectrum of 1:1 molar reaction mixture of PLP and **(A)** cysteine, **(B)** *S*-methylcysteine (SMC), and **(C)** *N*-acetylcysteine (NAC) in D_2_O reacted at 37 °C for 2 h. Schiff base is formed with the reaction of PLP and SMC. The aldiminic proton of a Schiff base is observed at δ ≈ 8 ppm. Hemimercaptal is formed with the reaction of PLP and NAC. The enolic proton of the hemimercaptal is observed at δ ≈ 6.5 ppm. Presence of Schiff base, hemimercaptal, and thiazolidine ring (δ = 6.05, 6.10 ppm) is observed with reaction of PLP and Cysteine.

Different intermediates of PLP reaction with cysteine are hypothesized in the literature^13–17^, which includes the formation of a Schiff base, hemimercaptal, and closed ring thiazolidine structure (Fig. 2A). These different potential PLP-Cys products were investigated by ^1^H-NMR and UV–Vis spectroscopy.

In the case of ^1^H-NMR spectroscopy, PLP and cysteine were reacted in deuterated water (D_2_O) at 37 °C in a 1:1 molar ratio for 2 h. The zoomed version of the ^1^H-NMR spectrum is shown in Fig. 2A (full spectrum in Fig. S1). PLP exhibits keto-enol tautomerism above pH 5 through its aldehyde moiety whose α-hydrogen (4) is observed at δ = 10.4 ppm and that of the enol form of PLP (4′) is observed at δ = 6.45 ppm (Fig. S2)^23^. PLP upon reaction with cysteine exhibits two new peaks that appear at δ = 6.05 ppm and 6.10 ppm corresponding to the methine proton (z) of the racemic mixture of the thiazolidine ring formed with PLP and cysteine^24^. A third new peak (x) was observed at δ = 8.09 ppm which may correspond to the aldiminic proton of the Schiff base formed between PLP and cysteine. No peaks corresponding to the intermediate products were observed when PLP and cysteine were reacted at a molar ratio of 1:10, instead, peaks for thiazolidine ring were observed (Fig. S3). To confirm the position of the aldiminic proton, and thus the formation of Schiff base, PLP and *S*-methylcysteine (SMC), a cysteine analog that does not have a free thiol for ring closure, were reacted similarly. This reaction produced a new peak (x) at δ = 8.05 ppm which corresponds to the aldiminic proton of the Schiff base formed between SMC and PLP (Fig. 2B, full spectrum in Fig. S4). SMC and PLP form a Schiff base within 2 h of reaction time as observed by the shift in the absorbance maximum in the UV–Vis spectrum of the reaction mixture from 388 nm, transitions typically attributed to aldehyde group, to 401 nm (Fig. S5). Therefore, the peak appearing at δ ≈ 8 ppm for PLP-cysteine reaction is attributed to the presence of the Schiff base structure. Alternatively, the cysteine may also react with PLP through the formation of a hemimercaptal (Fig. 2A)^17^, which is possible by the interaction of electron lone pair on the sulfur with the enolic proton of the PLP. A small shoulder peak (y) was observed at δ = 6.49 ppm in Fig. 2A which hints towards the possibility of the formation of a hemimercaptal. To confirm the formation of a hemimercaptal, PLP and *N*-acetylcysteine (NAC), a cysteine analog which has free thiol for hemimercaptal formation but not a free amine for forming Schiff base, were reacted and one new peak (y) was observed at δ = 6.47 ppm which corresponds to the enolic proton of the hemimercaptal formed between NAC and PLP (Fig. 2C, full spectrum in Fig S6). No change in the intensity of the PLP peak at 388 nm in the UV–Vis spectrum was observed after reacting NAC and PLP for 2 h (Fig. S7). To ascertain that the secondary amine from NAC does not form Schiff base producing the new peak at δ = 6.47 ppm, PLP and *N*-acetylmethionine (NAM), a cysteine analog without free amine or thiol, were reacted and no new peaks were observed after 2 h of reaction confirming the position of the hemimercaptal proton at δ ≈ 6.47 ppm (Fig. S8). Based on these observations, we can propose that cysteine and PLP reaction in water results in formation of all three products: thiazolidine ring structure, Schiff base, and hemimercaptal.

The interaction of PLP with cysteine in physiological conditions of ionic strength and temperature was determined with absorption spectroscopy. PLP and cysteine were reacted in phosphate-buffered saline (PBS) at 37 °C for 2 h. To determine the potential reaction products from experiments, the UV–Vis spectrum for potential products was calculated computationally (xyz coordinates for structures in Fig. S9) with hybrid density functional theory.

**Figure 3:**
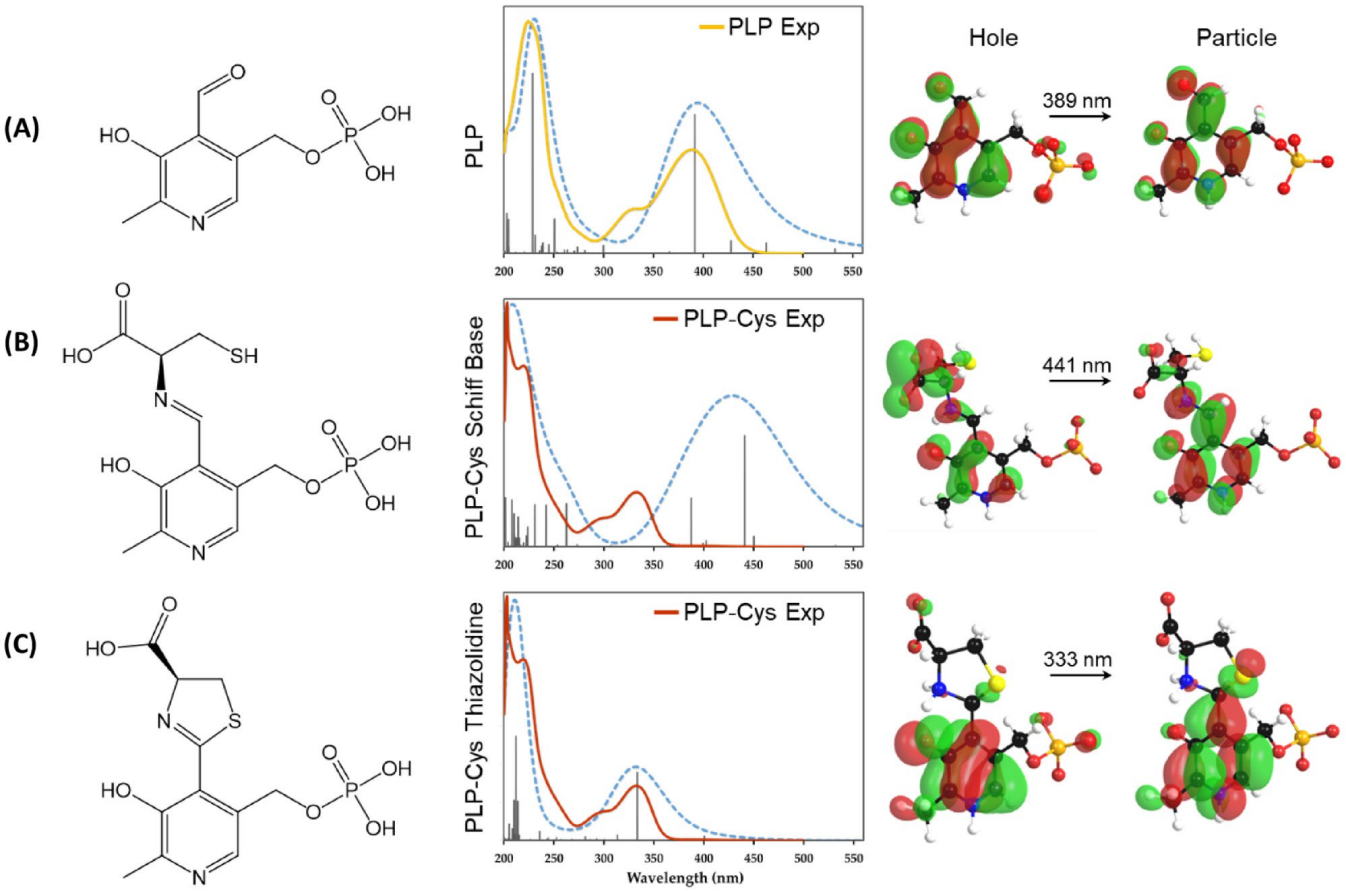
Comparison of experimental (solid lines) and calculated (dashed blue lines) absorption spectra for **(A)** PLP, **(B)** PLP-cysteine Schiff base, and **(C)** PLP-cysteine thiazolidine ring structures. The left side of each panel shows the molecular structure and the right side of each panel shows the natural transition orbitals at the peak of interest. The experimental spectrum of PLP matches well with calculated spectrum. The experimental spectrum of PLP-cysteine reaction after 2 h in PBS matches with formation of thiazolidine structure and not with Schiff base.

The calculated PLP absorption spectrum (Fig. 3A) is characterized by a strong optical response in the UV range with dominant peaks at 228 nm and 392 nm. Less intense peaks appear at 205 nm and 250 nm. The HOMO–LUMO transition for PLP is calculated to be 2.33 eV or 532 nm corresponding to a transfer of electron density from the phosphate group to the aromatic group (Fig. S10A). At the peak of interest (392 nm), the natural transition orbitals show the re-distribution of electron density states in the aromatic group (Fig. 3A). The experimental spectrum for PLP solution in PBS exhibits two dominant peaks at 225 nm and 388 nm, which matches well with the calculated spectrum (Fig. 3A). The experimental spectrum also has a shoulder peak at 328 nm, which is absent in the calculated spectrum. This additional peak could be attributed to additional zwitterionic equilibrium states of PLP^25–27^ Besides the shoulder peak, the experimental spectrum is well reproduced in calculations. Since PLP can exhibit keto-enol tautomerism through its aldehyde moiety above pH 5, the UV–Vis spectrum for the enol form of PLP was also calculated (Fig. S11). The calculated spectrum has dominant peaks at 451 nm, 290 nm, and 223 nm. The calculated peaks do not match with experimental data and, thus, suggest that PLP exists only as an aldehyde form in physiological conditions of ionic strength and temperature.

The calculated absorption spectrum for PLP-Cys Schiff base (Fig. 3B) exhibits dominant peaks at 441 nm and 387 nm, which together produce a Gaussian peak at 428 nm. Several less intense peaks in the range of 201–262 nm enhance the broad Gaussian peak at 203 nm. The HOMO–LUMO transition for PLP-Cys Schiff base structure is calculated to be 1.46 eV or 849 nm corresponding to a transfer of electron density from the phosphate group to the aromatic group and Schiff base connected cysteine (Fig. S10B). At the peak of interest (441 nm), the natural transition orbitals show the re-distribution of electron density states in the aromatic group as well as transfer from the carboxylic acid group to the aldimine structure (Fig. 3B). Similarly, the calculated absorption spectrum for the PLP-Cys thiazolidine structure (Fig. 3C) exhibits dominant peaks at 333 nm and 212 nm. Several less intense peaks in the range of 204–220 nm enhance the broad Gaussian peak at 210 nm. The HOMO–LUMO transition for PLP-Cys thiazolidine structure is calculated to be 2.49 eV or 497 nm corresponding to a transfer of electron density from the phosphate group to the aromatic and thiophene groups (Fig. S10C). At the peak of interest (333 nm), the natural transition orbitals show the re-distribution of electron density states in the aromatic group as well as transfer to the thiophene group (Fig. 3C).

The experimental spectrum for PLP cysteine mixture in PBS exhibits a broad peak at 203 nm with a shoulder peak at 220 nm and a second dominant peak at 333 nm with a shoulder peak at 294 nm. The presence of a peak at 333 nm and the absence of any absorption above 400 nm suggests that either the PLP and cysteine do not form a Schiff base or the Schiff base is unstable and rapidly cyclizes to form the ring^17^. Interestingly, the experimental data matches with the calculated PLP-Cys thiazolidine absorption spectrum. The shoulder peak at 294 nm is not predicted and could be attributed to shifted zwitterionic equilibrium states of PLP.

The results from ^1^H-NMR and UV–Vis spectroscopy along with calculated absorption spectra confirm the presence of the thiazolidine ring with PLP-Cys reaction. Similarly, the formation of hemimercaptal is supported by ^1^H-NMR spectroscopy. However, it is interesting to note that the Schiff base aldiminic structure is observed with NMR spectroscopy where the solvent was D_2_O, but not with UV–Vis spectroscopy where the solvent was PBS. This could be due to differences in the concentration of reactants, solvent ionic strength, or pH under which the Schiff base is unstable and rapidly cyclizes to form the thiazolidine ring^17^. Different concentrations of PLP were employed because a PLP concentration below 10 mM is not detectable in the ^1^H-NMR spectrum, and concentration above 0.1 mM saturated the UV–Vis signal at 388 nm, which is the peak of interest. To verify the formation of different intermediates between PLP and cysteine, reaction mixtures of PLP and cysteine in deionized water and PBS were analyzed with LC/MS. Very low abundance of intermediate ions of masses consistent with PLP-Cys Schiff base, thiazolidine ring and hemimercaptal were observed (Fig. S12). The signal for PLP-Cys Schiff base was not significantly higher than control. Although the presence of these intermediates is consistent with NMR and UV–Vis spectroscopy, it should be noted that the detected LC/MS signals were not strong enough to confirm the formation of these intermediates. The intermediates may not be stable in the column or gas phase during MS analysis, especially since the mobile phase was acidic: 0.1% formic acid (95% in Water and 5% in Acetonitrile).

**Figure 4:**
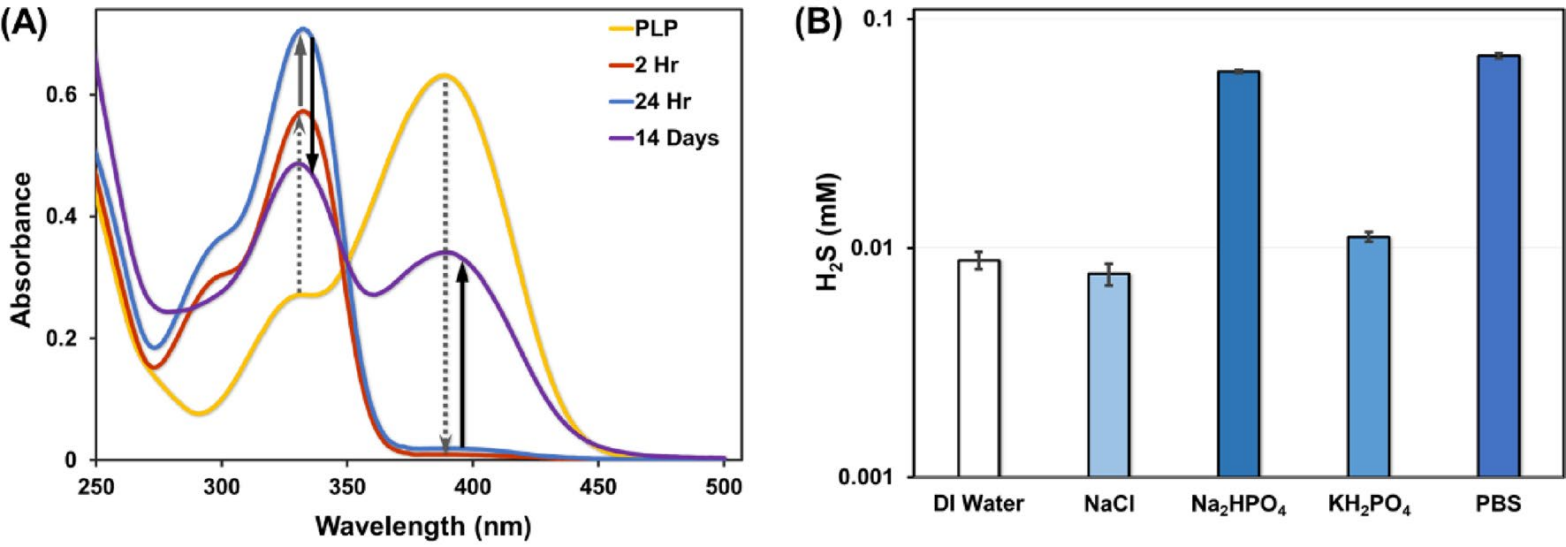
**(A)** UV–Vis spectrum of the chemical interaction between PLP and cysteine at physiological conditions monitored at 0 h, 2 h, 24 h, and 14 days. **(B)** H_2_S production from the reaction of PLP and cysteine in DI water, individual components of PBS, and PBS at 37 °C and 24 h. H_2_S production is 9 times more in PBS than DI Water.

The time-dependent interaction of PLP with cysteine was studied using UV–Vis spectroscopy. PLP and cysteine were reacted in PBS at 37 °C in the molar ratio of 1:10 for 14 days. Their absorbance spectrum was obtained to monitor the reaction (Fig. 4A). The absorbance spectrum for PLP at pH = 7.2 shows a maximum at 388 nm as discussed above^28^. After the addition of cysteine, the absorption at 388 nm decreases immediately suggesting formation of hemimercaptal and thiazolidine structures^17^. This peak completely disappears in 2 h suggesting that the aldehyde group of PLP has completely reacted with the cysteine and formed a thiazolidine ring with a peak increase observed at 333 nm. The UV–Vis spectrum shows the same trend until 24 h of reaction time with stronger absorption for the thiazolidine ring, which is in agreement with the literature^15^. However, at 24 h a small peak appears at 388 nm, corresponding to the aldehyde group of PLP. The intensity of the peak at 388 nm increases with time (7 and 14 days) with a concomitant decrease in the thiazolidine peak at 333 nm. This reversal suggests that the thiazolidine ring is being converted back to its PLP form. *S*-(*p*-substituted phenyl) analogs were observed as the by-product when *S*-(*p*-substituted phenyl) cysteines were reacted with PLP in slightly alkaline conditions regenerating the PLP^18^. Therefore, we propose that PLP functions as a weak catalyst for catabolizing cysteine in the absence of metal ions to form hydrogen sulfide (H_2_S).

**Figure 5:**
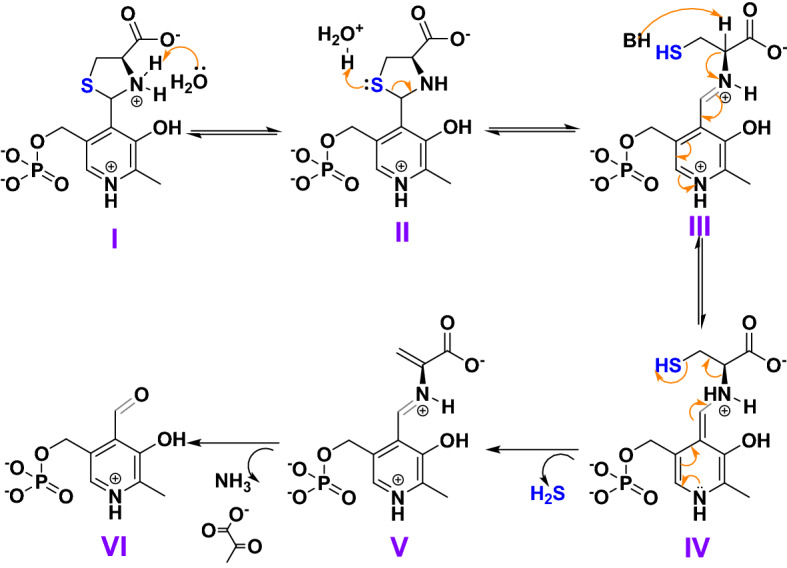
Proposed plausible reaction mechanism for enzyme-independent PLP catalyzed breakdown of cysteine to produce H_2_S at physiological conditions in the absence of metal.

To verify the ability of PLP to catalyze cysteine breakdown to produce H_2_S, we performed paper chromatography (lead acetate assay) to detect the production of H_2_S gas as one of the by-products. It must be noted that less than 20% of H_2_S produced is in gas phase at physiological pH^29,30^. The reaction of PLP and cysteine at physiological conditions in PBS generated nearly 70 µM H_2_S in 24 h (Fig. 4B). No H_2_S production was observed in the absence of PLP (Fig. S13A). Interestingly, H_2_S evolution was 8.8 µM when the reaction was performed in de-ionized (DI) water (Fig. 4B). PBS has a pH of 7.4 and is composed of 137 mM sodium chloride (NaCl), 2.7 mM potassium chloride (KCl), 8 mM disodium hydrogen phosphate (Na_2_HPO_4_), and 2 mM potassium dihydrogen phosphate (KH_2_PO_4_). The reaction of PLP and cysteine in presence of each of the salts individually generated nearly 7.6 µM H_2_S with 137 mM NaCl, 58.9 µM H_2_S with 8 mM Na_2_HPO_4_, and 11.1 µM H_2_S with 2 mM KH_2_PO_4_, in 24 h. These results suggest that the presence of Na_2_HPO_4_ in PBS is a major contributor to H_2_S production. It may be because Na_2_HPO_4_ in water is moderately basic (pH 8.86) and releases a hydroxide ion forming phosphoric acid. The released hydroxide ion may act as a base in extracting the α-proton from the PLP-Cys Schiff base (discussed in Fig. 5) and pushing the reaction forward to produce H_2_S. Since PLP catalyzed cysteine breakdown is accelerated in basic medium^18^, the H_2_S production is expected to increase in the presence of slightly alkaline conditions. In addition, PLP solution in alkaline conditions exhibit a deeper yellow color as seen with PLP solution in PBS compared with PLP solution in DI water (Fig. S13B)^28^.

After 14 days of reaction time, the absorbance peak of the thiazolidine group at 333 nm decreases and that of the aldehyde group at 388 nm increases proportionally. This suggests that PLP, along with producing H_2_S at physiological conditions, is capable of slowly regenerating itself to behave as a catalyst. These results demonstrate that PLP can function as a catalyst for the breakdown of cysteine to produce H_2_S under physiological conditions. Most importantly, we show, for the first time, that PLP catalyzes cysteine breakdown in the absence of (i) enzymes, (ii) base, and (iii) metal ions to produce H_2_S.

Based on our results and the literature, we propose a plausible reaction mechanism for PLP catalyzed cysteine break down in PBS to produce H_2_S as presented in Fig. 5. Since the PLP and cysteine reaction is complete within 2 h to form a thiazolidine ring, the H_2_S producing pathway must proceed through the ring-opening mechanism. In the absence of enzymes, base, and metal ions, the thiazolidine ring may open via hydrolysis (Fig. S14)^31^. As presented in Fig. 5, the ring hydrolysis may proceed via the attack of water on the positively charged nitrogen group of **I** to generate the hydronium ion that attacks the sulfur on the ring on **II** causing the ring to open and probably form a Schiff base **III**. Since the Schiff base is highly unstable^17^, water molecules or weak bases may abstract the α-proton from the activated methine of cysteine from **III** and generate hydronium ion and a quinonoid structure **IV**. This quinonoid structure can then stabilize itself by β-elimination of the thiol group to form **V** and generate H_2_S. The structure formed **V** may then decompose to form ammonia and pyruvate to regenerate the PLP **VI**. These reaction intermediates or by-products were not observed with ^1^H-NMR or UV–Vis spectroscopy and will require further investigation.

We have shown for the first time that PLP catalyzed cysteine breakdown in the absence of (i) enzymes, (ii) base, and (iii) metal ions to produce H_2_S at physiological conditions. PLP completely reacts with cysteine to form thiazolidine ring within 2 h of reaction. The ring formation was observed with ^1^H-NMR and UV–Vis spectroscopy. The computationally obtained UV–Vis spectra helped in confirming formation of thiazolidine ring structure between PLP and cysteine. Direct observation of the Schiff base and hemimercaptal formation between cysteine and PLP has not been reported previously. The thiazolidine ring slowly decomposes to generate 70 µM H_2_S within 24 h. Interestingly, less H_2_S (8.8 µM) was produced in de-ionized water, suggesting a dominant role of ionic strength and pH. Beyond 24 h, evidence of regeneration of PLP is observed with UV–Vis spectroscopy. This suggested that PLP, along with producing H_2_S at physiological conditions, is capable of slowly regenerating itself to behave as a catalyst. Based on the spectroscopic and computational evidence provided, a plausible reaction mechanism was proposed for PLP catalysis. This chemical investigation of enzyme-independent PLP catalysis is important due to its biological relevance in producing H_2_S, which is an essential nutrient for the body and its endogenous deficiency is associated with several detrimental health effects.

## Experimental

### Materials

Pyridoxal-5′-phosphate hydrate (PLP), L-cysteine (Cys), disodium hydrogen phosphate (Na_2_HPO_4_), and potassium dihydrogen phosphate (KH_2_PO_4_) were obtained from Alfa Aesar (MA, USA). Deuterium oxide (D_2_O), *N*-acetylcysteine (NAC), *N*-acetylmethionine (NAM), sodium chloride (NaCl), and lead (II) acetate trihydrate were obtained from Sigma-Aldrich (MO, USA). *S*-methylcysteine (SMC) was obtained from TCI America (OR, USA). All chemicals were used without further purification.

### Methods

*Nuclear magnetic resonance (NMR) spectroscopy* 20 mM PLP and 20 mM Cys in D_2_O were mixed in an NMR tube and placed in an incubator at 37 °C. ^1^H-NMR spectroscopy was used to monitor the reaction products. Bruker 400 MHz spectrometer (MA, USA) was used to record the ^1^H-NMR spectra in D_2_O with 16 scans. A similar experiment was performed using 20 mM PLP and 20 mM SMC, 20 mM NAC, 20 mM NAM respectively to monitor their reaction products.

*UV–Vis spectroscopy* 0.1 mM PLP and 1 mM Cys in PBS (pH = 7.2) pre-heated to 37 °C were mixed in a quartz cuvette. The ground-state absorption spectrum of the reaction products was obtained with a Perkin Elmer Lambda 1050 UV–Vis-NIR spectrophotometer (Waltham, MA) and temperature controlled cuvette holder at 37 °C.

*Paper chromatography assay for H*_*2*_*S measurement* 1 mM PLP and 10 mM Cys in DI Water were reacted in a 96-well polypropylene plate in an incubator at 37 °C for 24 h. A similar experiment was performed with reactants prepared in 137 mM NaCl, 8 mM Na_2_HPO_4_, 2 mM KHPO_4_, and PBS. The gaseous H_2_S evolved from the reaction was quantified using lead acetate paper assay. The lead acetate paper assay and its analysis were performed as reported previously^32^.

*Computational study on PLP-cysteine interactions* All computations for this study were performed with the General Atomic and Molecular Electronic Structure System (GAMESS) 2018-R1 code^33^ using density functional theory (DFT) and hybrid B3LYP functional^34–37^. A standard polarizable continuum model (PCM) with water as solvent was employed for all calculations. Initial Cartesian coordinates of molecules were obtained with Avogadro (an open-source molecular builder and visualization tool)^38^ and the ground state molecular structures were optimized with a 6-311G(d, p) basis set. Time-dependent density functional theory (TDDFT) calculations for the geometry optimized molecules were run with closed-shell restricted Hartree–Fock (RHF) wavefunctions. The natural transition orbitals, as well as spectra presenting the oscillator strength as a function of wavelength (excitation energy) and the curve obtained by a Gaussian broadening (with a full width of half-height of 0.08 eV), were acquired with Chemissian software.

*Liquid chromatography-mass spectroscopy* Liquid chromatography-Mass spectroscopy (LC–MS) was carried out at the Proteomics and Metabolomics core facility, Lerner Research Institute. Untargeted metabolomics using reverse phase chromatography was performed by injecting 4 µL of each sample on a Thermo Accucore Vanquish C18 column with dimensions 100 × 2.1 mm, 1.5 µm particle size (Thermo P/N 27101‐102130) at 60 °C coupled to a Thermo Vanquish UHPLC by gradient elution where mobile phase A is 0.1% formic acid in water and mobile phase B is 0.1% formic acid in acetonitrile. The Orbitrap Q Exactive HF was operated in positive and negative electrospray ionization modes in different LC‐MS runs over a mass range of 56–850 Da using targeted selected ion monitoring (t‐SIM) with MS1 at 120,000 resolution.

## Supplementary Information

Supplementary Information.

## Data Availability

The authors declare that all other data supporting the findings of this study are available within the paper and supplementary information.
