# Supplementary material for: Enzyme-independent catabolism of cysteine with pyridoxal-5′-phosphate
Source: Sci Rep. 2023 Jan 6;13:312. doi: 10.1038/s41598-022-26966-6 (PMC9822980; doi:10.1038/s41598-022-26966-6)
Supplement: Supplementary file 1 — Supplementary Information. [file 41598_2022_26966_MOESM1_ESM.docx]

**SUPPORTING INFORMATION**

**Enzyme-Independent Catabolism of Cysteine with Pyridoxal-5’-Phosphate**

**Prajakatta Mulay^1^, Cindy Chen^1^, and Vijay Krishna^1, 2*^**

*^1^Department of Biomedical Engineering, Lerner Research Institute, Cleveland Clinic, Cleveland, OH 44195, ^2^Department of Biomedical Engineering, Cleveland Clinic Lerner College of Medicine, Case Western Reserve University, Cleveland, OH 44106*

**Email: krishnv2@ccf.org*

**Table of Contents**

| **Figure** | **Description** | **Page** |
| --- | --- | --- |
| S1 | ^1^H-NMR full spectrum of 1:1 molar mixture of PLP and cysteine | S2 |
| S2 | ^1^H-NMR spectrum of PLP | S3 |
| S3 | ^1^H-NMR spectrum of 1:10 molar mixture of PLP and cysteine | S4 |
| S4 | ^1^H-NMR spectrum of 1:1 molar mixture of PLP and *S*-methylcysteine (SMC) | S5 |
| S5 | UV-Vis spectrum of the chemical interaction between PLP and *S*-methylcysteine (SMC) | S6 |
| S6 | ^1^H-NMR spectrum of 1:1 molar mixture of PLP and *N*-acetylcysteine (NAC) | S7 |
| S7 | UV-Vis spectrum of the chemical interaction between PLP and *N*-acetylcysteine (NAC) | S8 |
| S8 | ^1^H-NMR spectrum of 1:1 molar mixture of PLP and *N*-acetylmethionine (NAM) | S9 |
| S9 | XYZ of calculated structures for PLP, PLP-Cys Schiff Base, and PLP-Cys thiazolidine ring structure | S10 |
| S10 | Calculated absorption spectrum for PLP, PLP-Cys Schiff Base, and PLP-Cys thiazolidine ring structure | S11 |
| S11 | Comparison of calculated and experimental absorption spectra for PLP in aldehyde (keto) form and enol form | S12 |
| S12 | LCMS Analysis of of 1:1 molar mixture of PLP and Cysteine in PBS at 37°C | S13 |
| S13 | H_2_S production from cysteine in presence or absence of PLP in PBS. Photograph of 4 mM PLP solution in DI Water and PBS | S14 |
| S14 | Reaction mechanism for the hydrolysis of the PLP-cysteine thiazolidine ring structure | S14 |

**
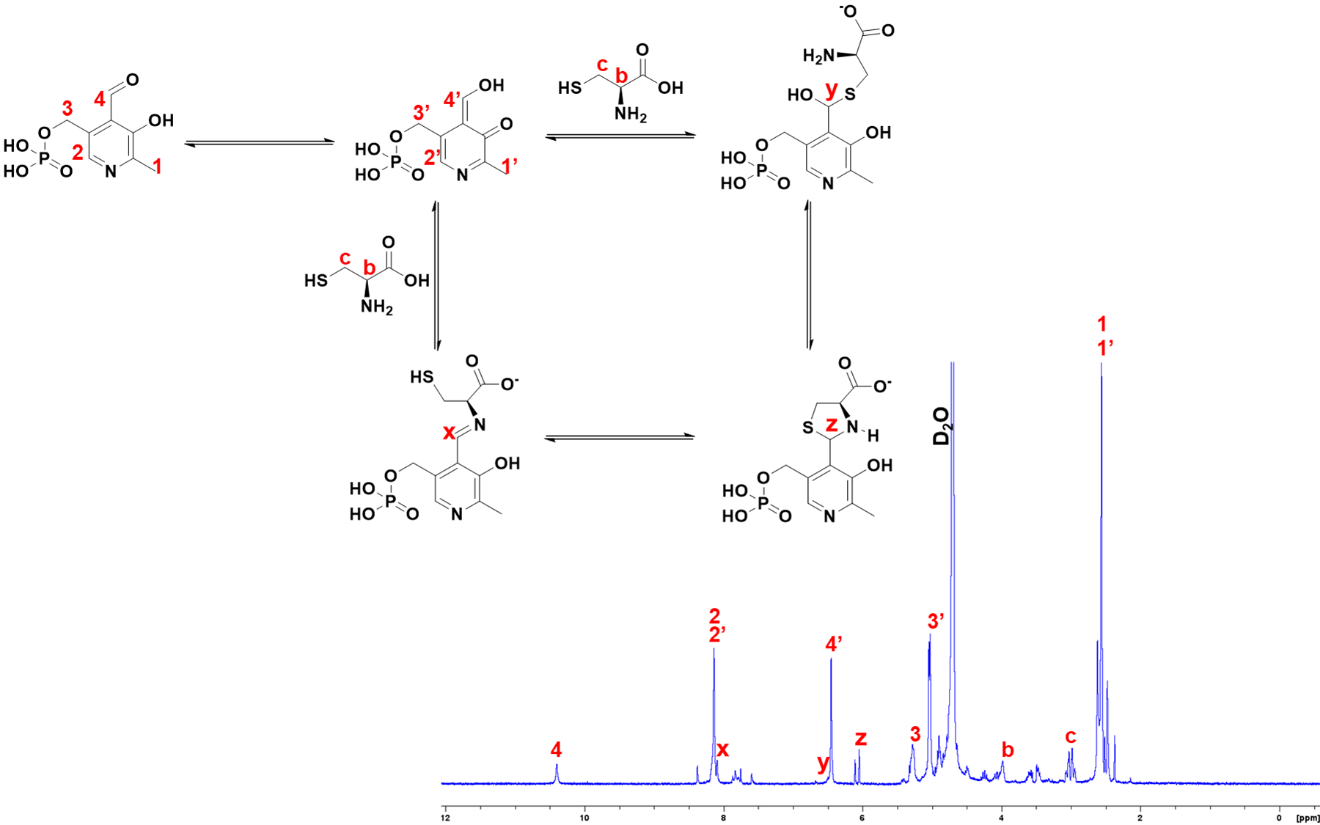
**

**Figure S1**: ^1^H-NMR full spectrum of 1:1 molar mixture of PLP and cysteine in D_2_O at 37°C. The aldiminic proton (x) of a Schiff base is observed at δ ≈ 8 ppm. The enolic proton (y) of the hemimercaptal is observed at δ ≈ 6.5 ppm. Presence of Schiff base, hemimercaptal and thiazolidine ring (δ ≈ 6.05, 6.10 ppm) is observed with reaction of PLP and cysteine.


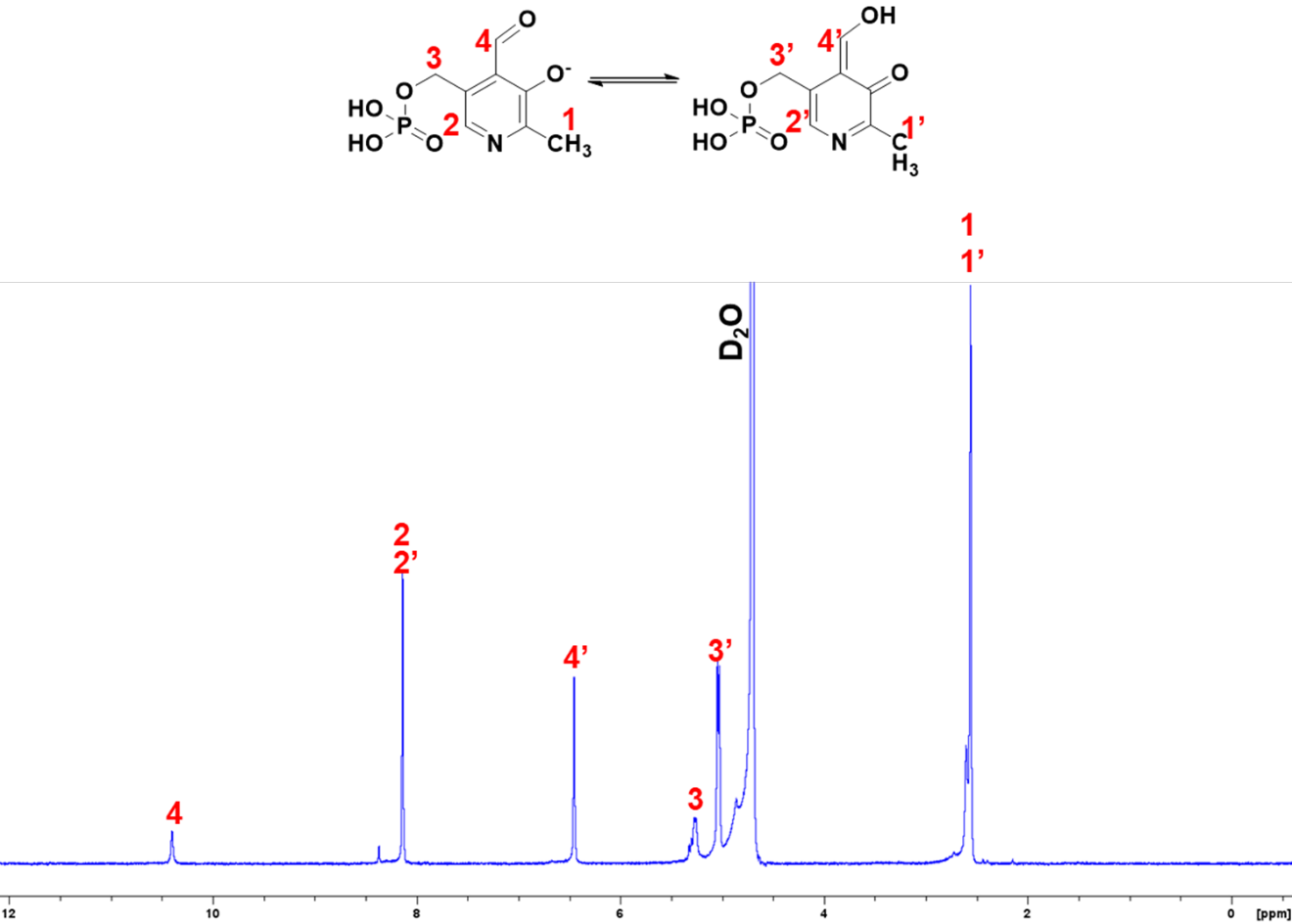


**Figure S2**: ^1^H-NMR spectrum of PLP. The enolic proton is observed at δ ≈ 6.45 ppm and the aldehyde proton is observed at δ ≈ 10.4 ppm.


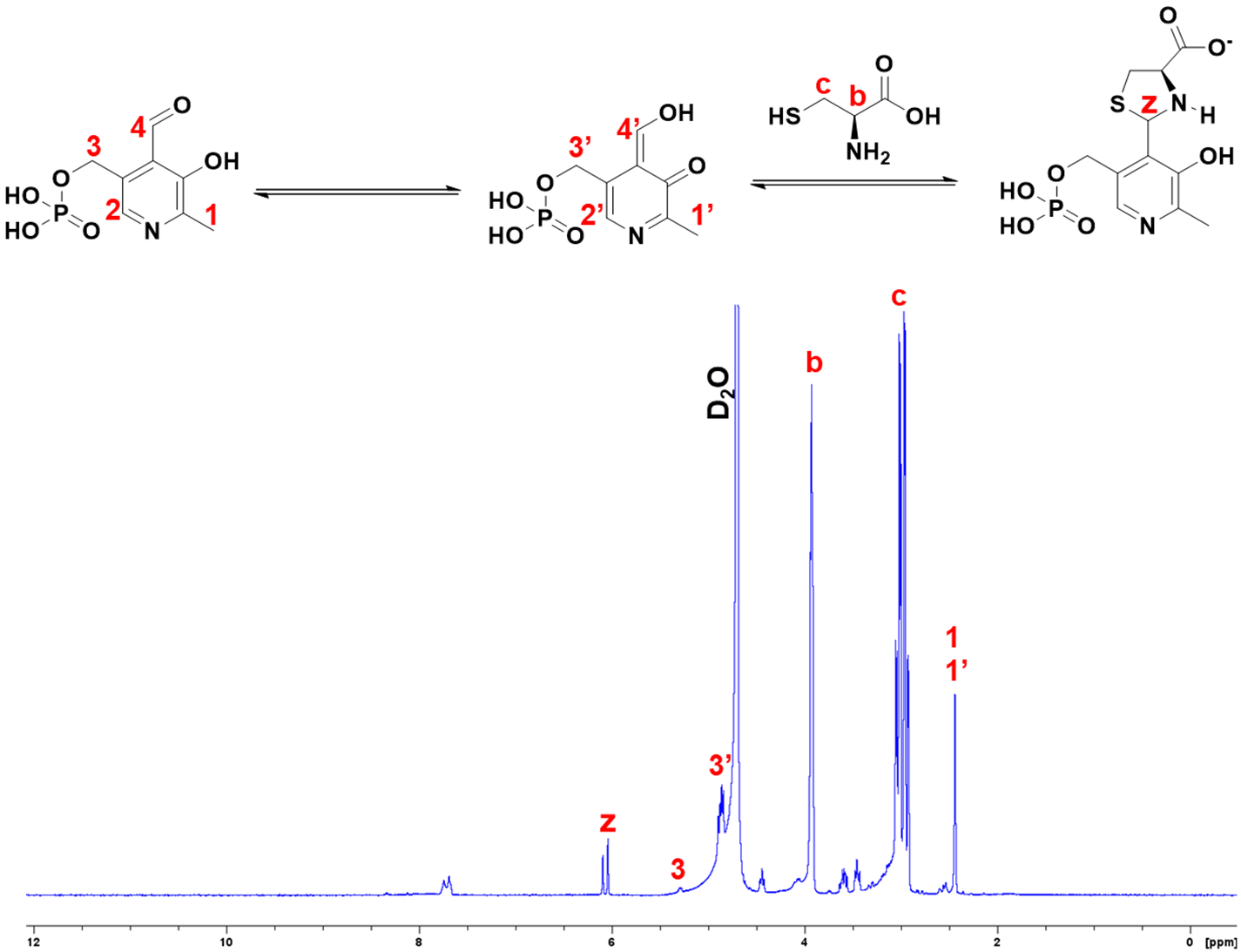


**Figure S3**: ^1^H-NMR spectrum of 1:10 molar mixture of PLP and cysteine in D_2_O at 37°C for 2 hours. Only thiazolidine ring (δ ≈ 6.05, 6.10 ppm) is observed with reaction of PLP and cysteine.


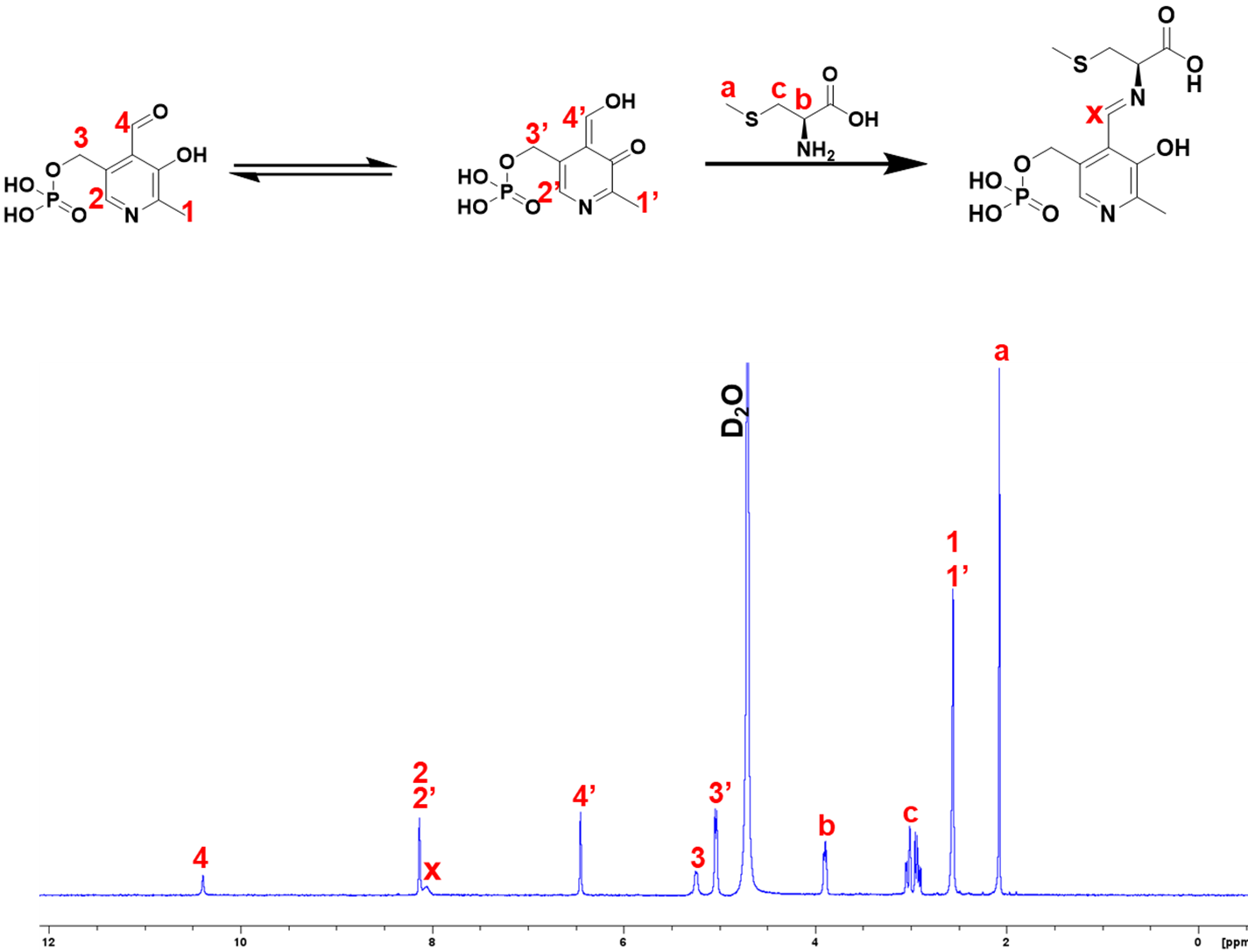


**Figure S4**: ^1^H-NMR spectrum of 1:1 molar mixture of PLP and *S*-methylcysteine (SMC) in D_2_O at 37°C for 2 hours. The reaction results in Schiff base and the aldiminic proton is observed at δ ≈ 8 ppm.


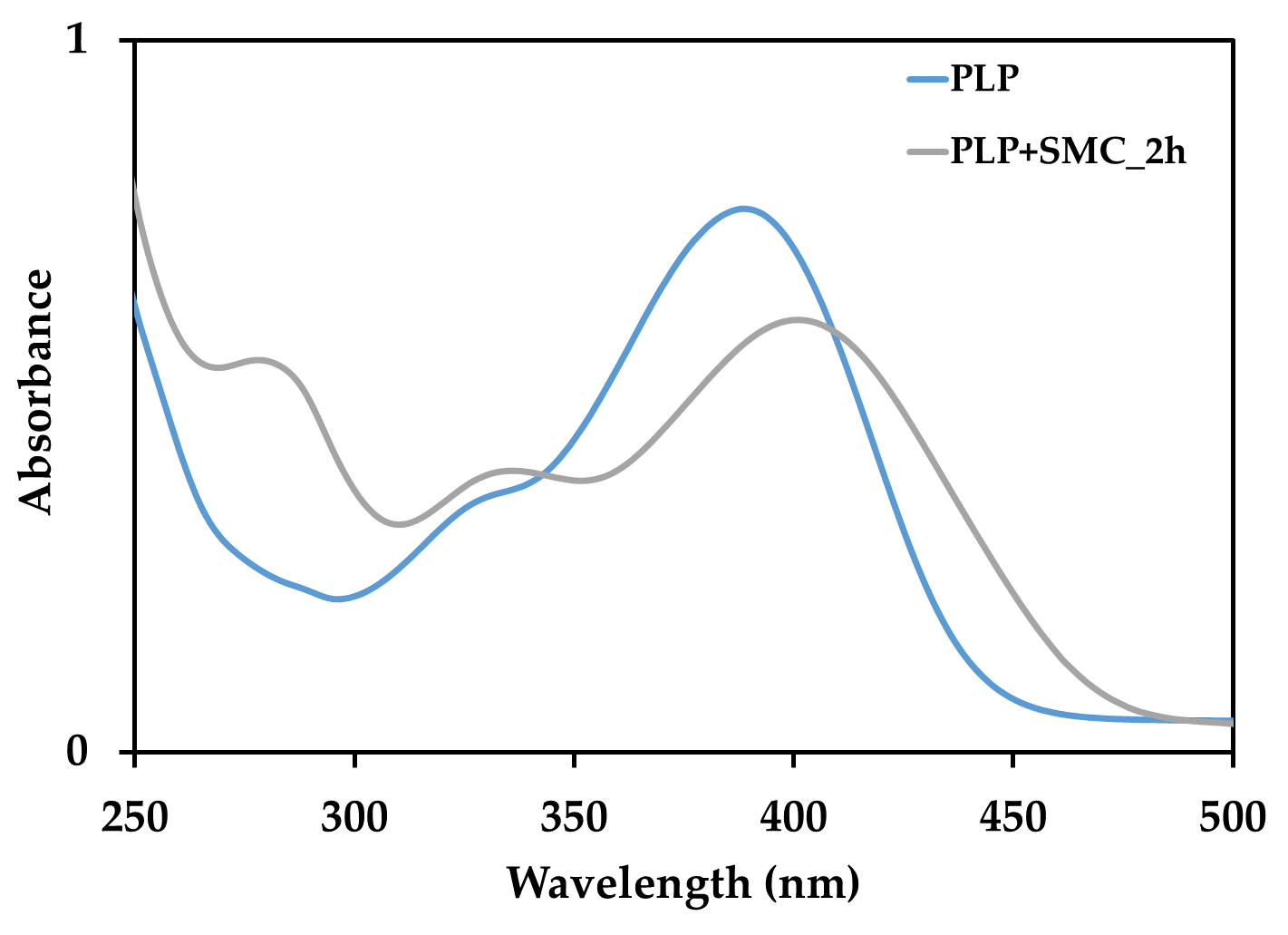


**Figure S5**: UV-Vis spectrum of the chemical interaction between PLP and *S*-methylcysteine (SMC) at physiological conditions monitored at 0 h and 2 h. Formation of Schiff base is observed with SMC within 2 hours with a peak shift from 388 nm to 401 nm.


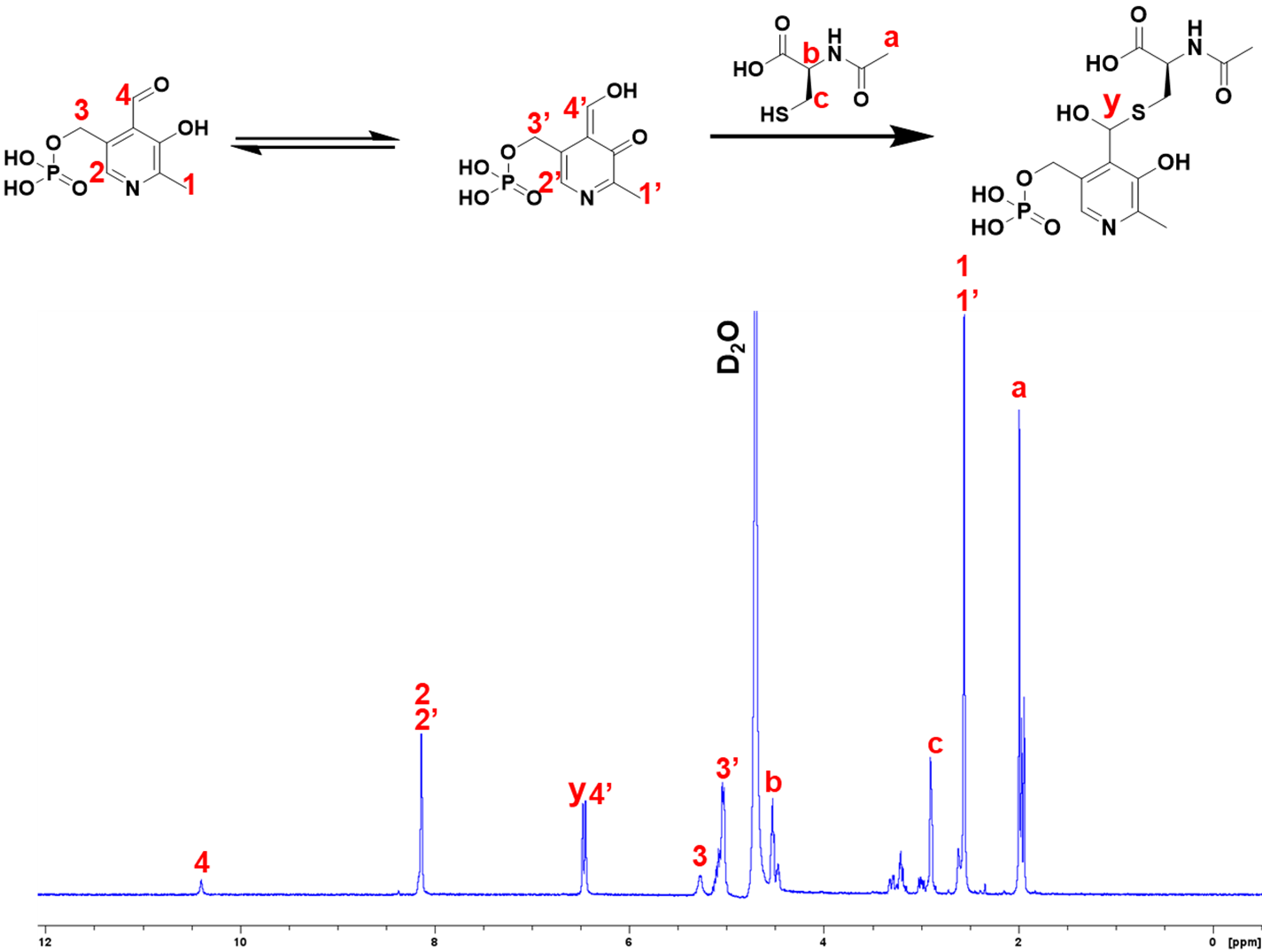


**Figure S6**: ^1^H-NMR spectrum of 1:1 molar mixture of PLP and *N*-acetylcysteine (NAC) in D_2_O at 37°C for 2 hours. The reaction results in hemimercaptal and the enolic proton is observed at δ ≈ 6.5 ppm.


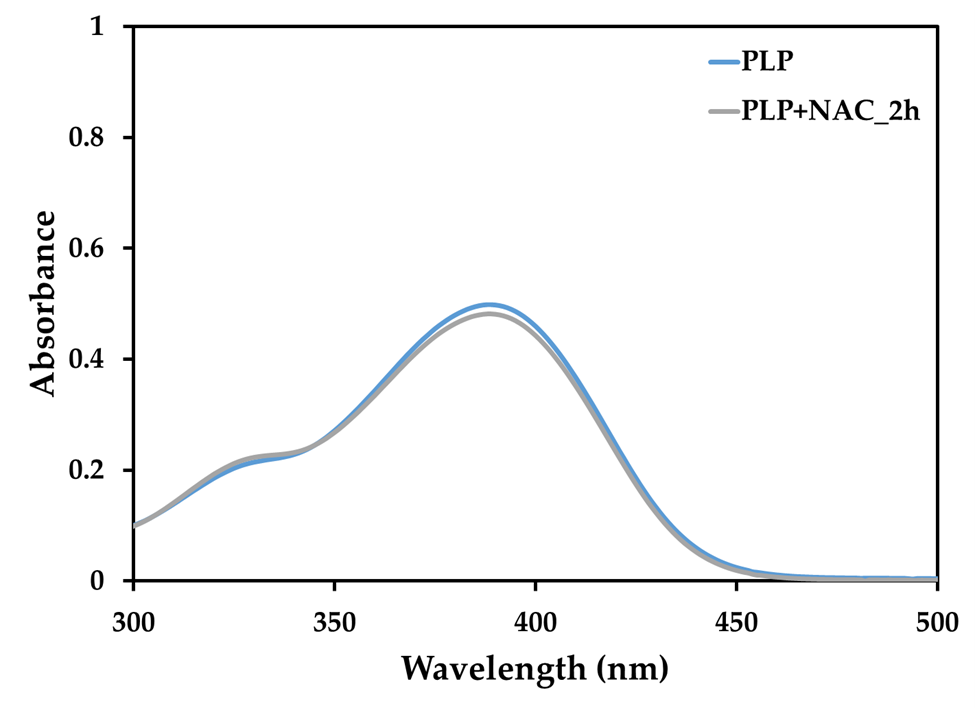


**Figure S7**: UV-Vis spectrum of the chemical interaction between PLP and *N*-acetylcysteine (NAC) at physiological conditions monitored at 0 h and 2 h. No change in peak position is observed with NAC within 2 hours.


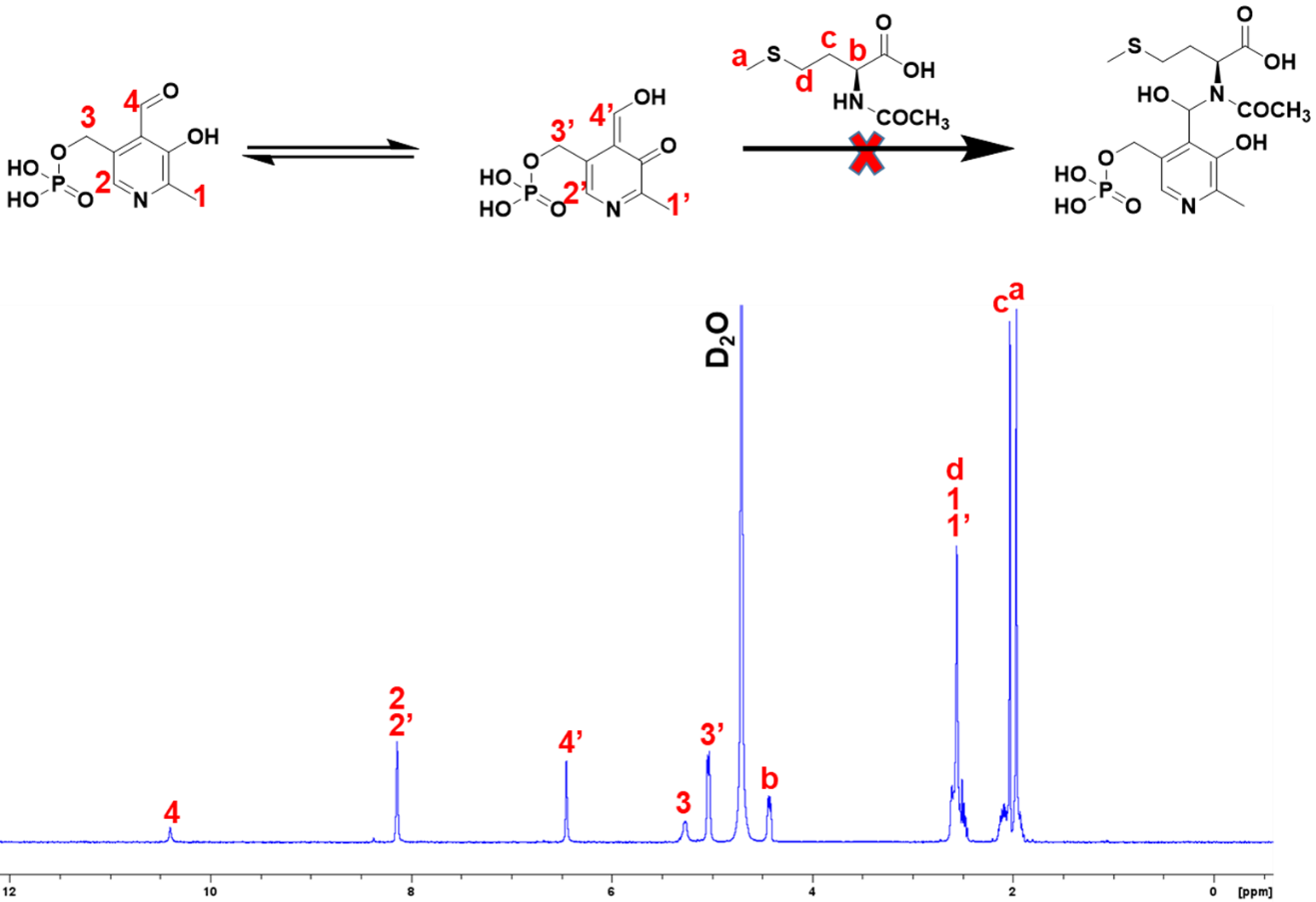


**Figure S8**: ^1^H-NMR spectrum of 1:1 molar mixture of PLP and *N*-acetylmethionine (NAM) in D_2_O at 37°C for 2 hours. No new peaks were present.


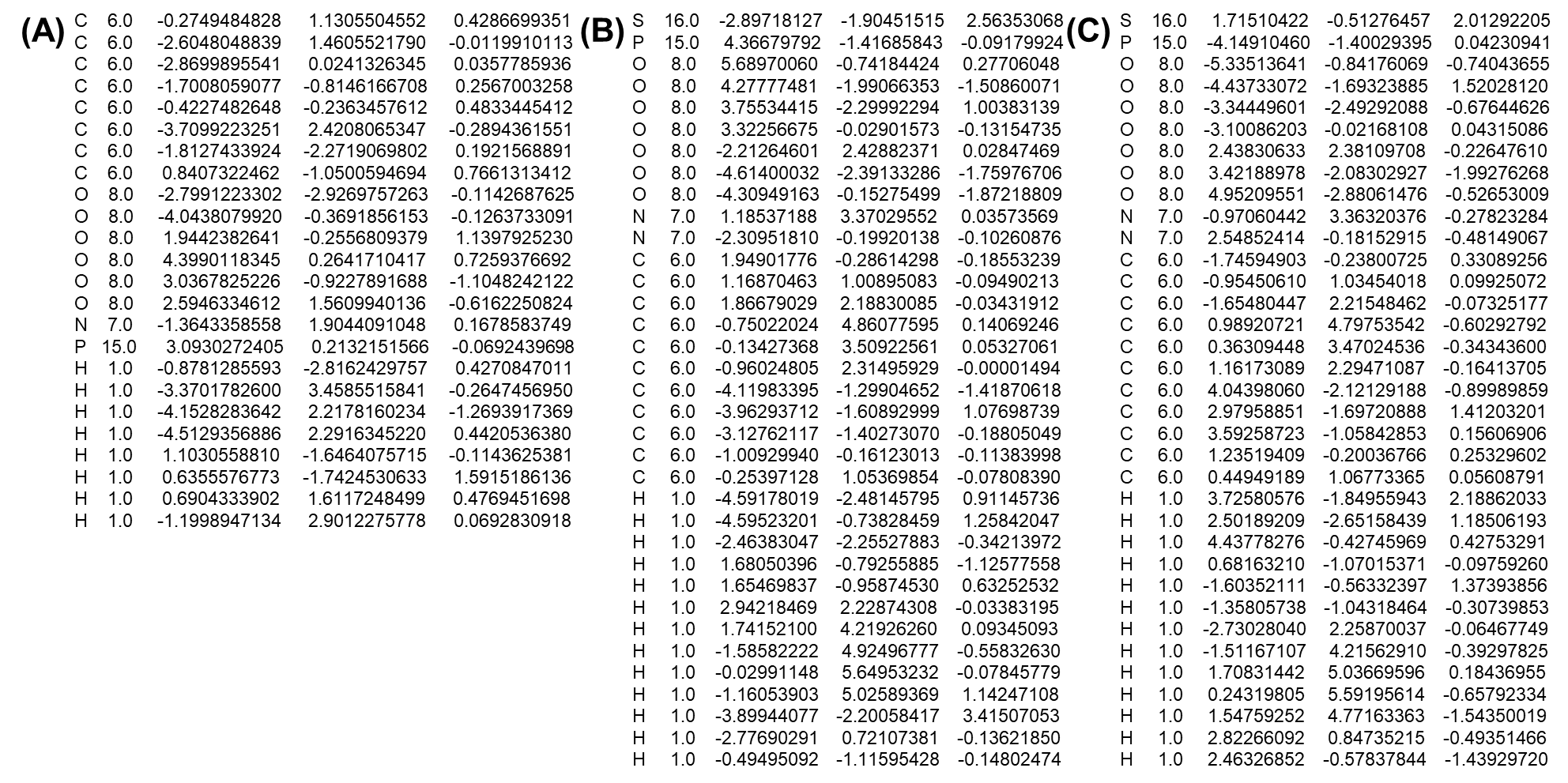


**Figure S9**: XYZ of calculated structures for **(A)** PLP, **(B)** PLP-Cys Schiff Base, and **(C)** PLP-Cys thiazolidine ring structure.


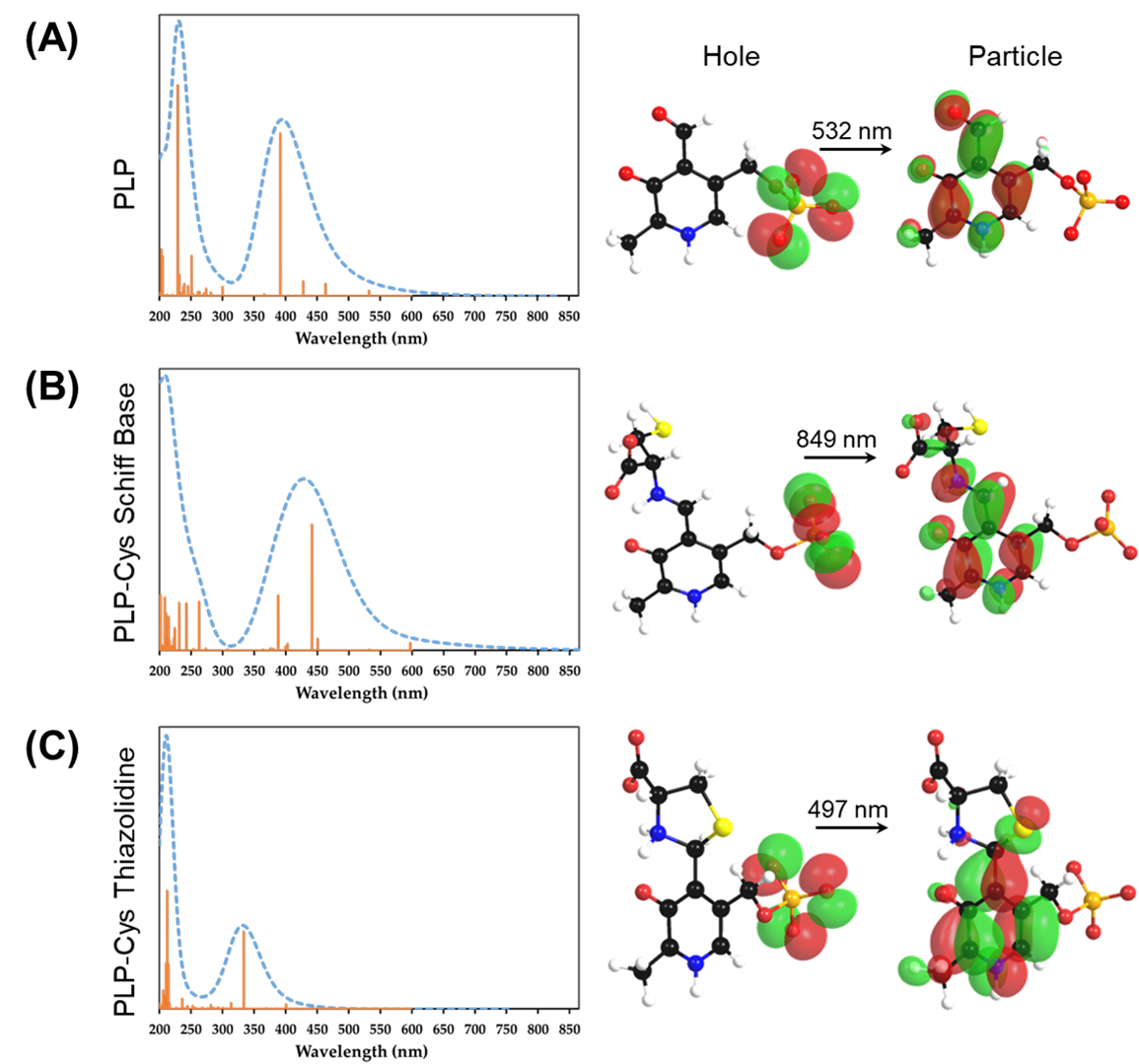


**Figure S10**: Calculated absorption spectrum for **(A)** PLP, **(B)** PLP-Cys Schiff Base, and **(C)** PLP-Cys thiazolidine ring structure. The HOMO-LUMO transitions are shown right of each spectrum.


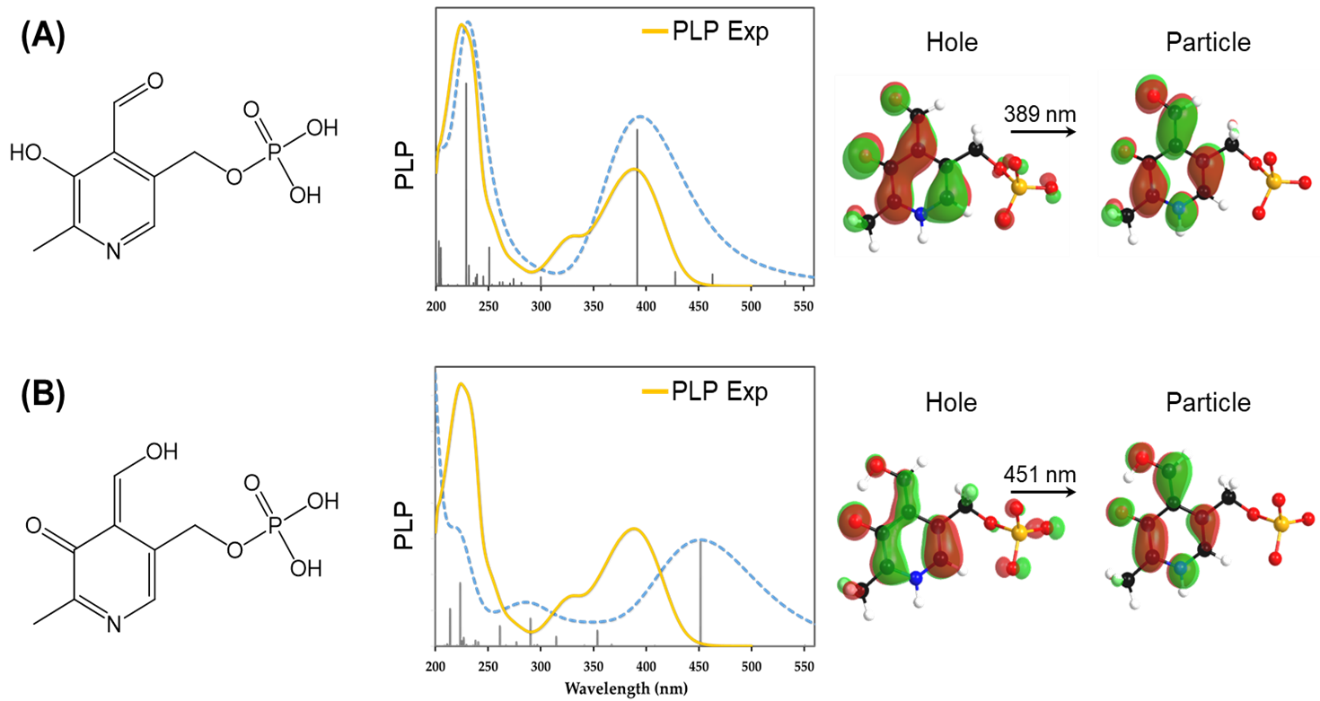


**Figure S11**: Comparison of calculated and experimental absorption spectra for **(A)** PLP in aldehyde (keto) form, and **(B)** PLP in enol form. The molecular structure and the natural transition orbitals at peak of interest are shown on left and right side of the absorbption spectrum, respectively.


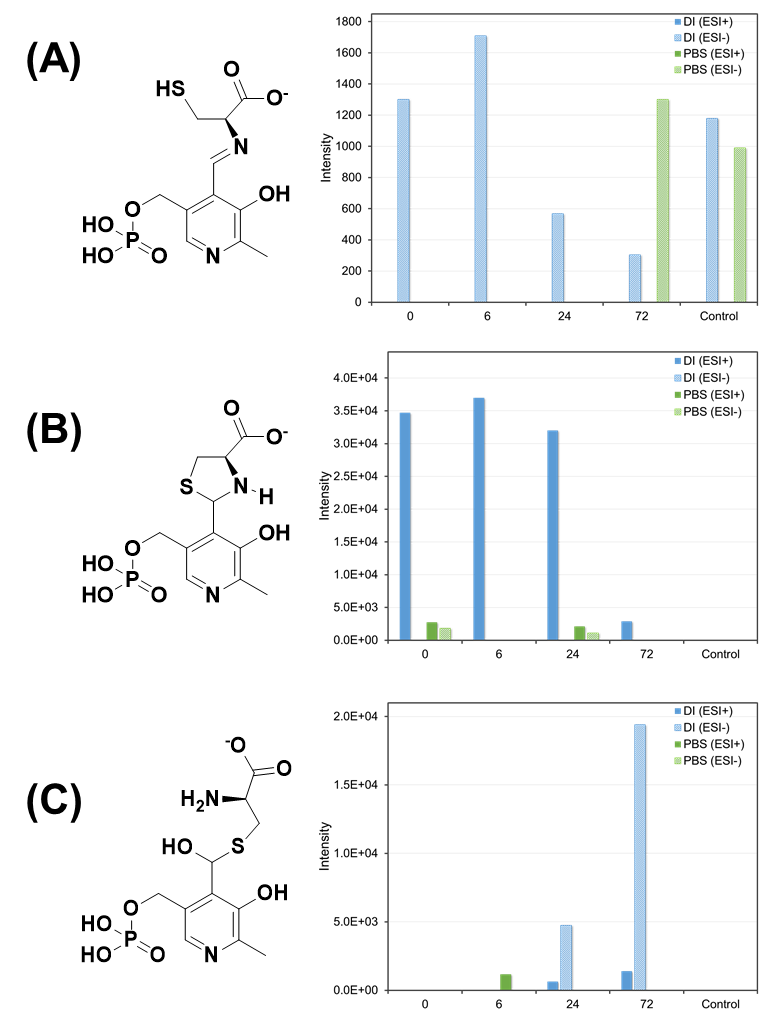


**Figure S12**: Abundance of **(A)** PLP-Cys Schiff base, **(B)** PLP-Cys thiazolidine ring and **(C)** PLP-Cys hemi-mercaptal ions in 1:1 molar mixture of PLP and cysteine in deionized (DI) water and PBS at 37°C after 0 h, 6 h, 24 h, and 72 h reaction time. PLP-Cys Schiff base intermediate signal was not significantly higher than control. PLP-Cys thiazolidine ring is relatively stable and was found at different time points in DI water and PBS media. PLP-Cys hemi-mercaptal was observed in DI water.


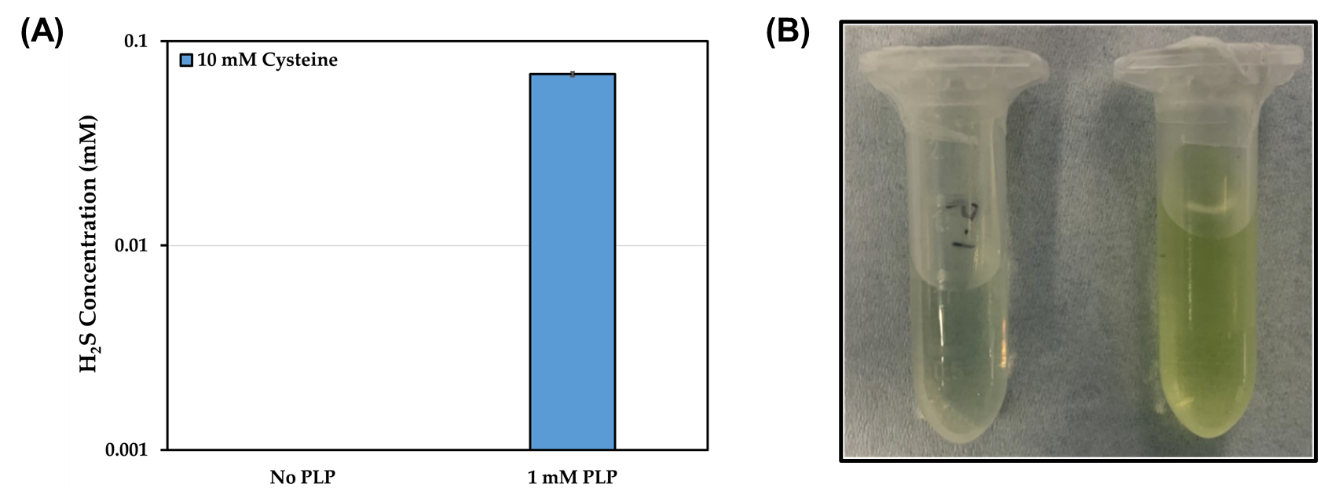


**Figure S13**: **(A)** H_2_S production from 10 mM cysteine in presence or absence of 1 mM PLP in PBS at 37°C for 24 hours. No H_2_S production was observed without PLP. **(B)** Photograph of 4 mM PLP solution in (left) DI Water and (right) PBS.

**Figure S14**: Reaction mechanism for the hydrolysis of the PLP-cysteine thiazolidine ring structure
